# Acquisition and Modeling of Material Appearance Using a Portable, Low Cost, Device

**DOI:** 10.3390/s25041143

**Published:** 2025-02-13

**Authors:** Davide Marelli, Simone Bianco, Gianluigi Ciocca

**Affiliations:** Department of Informatics, Systems and Communication, University of Milano-Bicocca, Viale Sarca 336, 20126 Milan, Italy; davide.marelli@unimib.it (D.M.); simone.bianco@unimib.it (S.B.)

**Keywords:** material acquisition, material measurement, material reproduction, low-cost device, photometric stereo, computer graphics

## Abstract

Material appearance acquisition allows researchers to capture the optical properties of surfaces and use them in different tasks such as material analysis, digital twins reproduction, 3D configurators, augmented and virtual reality, etc. Precise acquisition of such properties requires complex and expensive hardware. In this paper, we aim to answer the following research challenge: Can we design an accurate enough but low-cost and portable device for material appearance acquisition? We present the rationale behind the design of our device using consumer-grade hardware components. Ultimately, our device costs EUR 80 and can acquire surface patches of size 5 × 5 cm with a 40 pix/mm resolution. Our device exploits a traditional RGB camera to capture a surface using 24 different images, each photographed using different lighting conditions. The different lighting conditions are generated by exploiting the LED rings included in our device; specifically, each of the 24 images is acquired by turning on one individual LED at time. We also illustrate the custom processing pipelines developed to support capturing and generating the material data in terms of albedo, normal, and roughness maps. The accuracy of the acquisition process is comprehensively evaluated both quantitatively and qualitatively. Results show that our low-cost device can faithfully acquire different materials. The usefulness of our device is further demonstrated by a textile virtual catalog application that we designed for rendering virtual fabrics on a mobile apparatus.

## 1. Introduction

Material appearance acquisition is the task of characterizing the optical properties of a surface and is a topic of interest in both computer vision and computer graphics fields. This topic is also relevant in the context of Industry 4.0, where it can be exploited for the creation of digital catalogs, digital twins, or reverse engineering of existing materials since it allows for the reproduction of elements that mimic real-world surfaces in the virtual world. The ability to virtually replicate the appearance of a surface enables the provision of interactive catalogs of object materials in order to replace the materials of real objects in 3D configurators for the customization, design, and prototyping of new items.

The appearance of materials can be modeled by a function. In the case of opaque materials, this function is called the Bidirectional Reflectance Distribution Function (BRDF) [1], which models the amount of light energy reflected from any incoming direction into any outgoing direction for any given wavelength at a specific point *p* of a surface. It is thus a 4D function that maps any pair of directions over the upper unit hemisphere to a non-negative real number. The BRDF is intended to provide information about how light is scattered by the material at a single point on the surface, and it is commonly used to represent the material properties of uniform surfaces. However, in the real world, most objects are not made of uniform materials, and light is not scattered in the same way across the surface. The spatially varying bidirectional reflectance distribution function (SVBRDF) [2] models the reflectance by adding a dependency on the surface position, making it a six-dimensional function.

Physically measuring material properties requires expensive devices such as gonioreflectometers that have long acquisition times and produce large amounts of data. These devices must also be carefully calibrated to align the light emitters with the detector sensors. To overcome these limitations, image-based approaches have been proposed. For example, in [3], an object of known shape is photographed with a series of images, each capturing light reflected from parts of the surface while photogrammetry is used to measure the camera position. Another image-based technique is photometric stereo (PS) [4]. It is a method for recovering local surface shape and albedo from multiple images taken from the same viewpoint but under different illumination directions. More recently, deep-learning-based methods have emerged to solve the task of material appearance acquisition. However, these methods require a large amount of training data to obtain suitable methods for estimating (not measuring) the material properties.

With the need for easy-to-use tools and devices for material acquisition in mind, this paper presents the design and development of a portable, low-cost device for the acquisition of a planar surface’s material appearance, which can later be used to create realistic renderings of real surfaces.

Our device utilizes a conventional RGB camera to capture surface images under 24 distinct lighting conditions. These varying illumination captures are achieved using the integrated LED rings in the device, with each image acquired by activating a single LED at a time. A specifically designed processing pipeline then measures the material appearance by computing the normal map, albedo map, and roughness map that can be used in a computer graphics software to render and reproduce the original material. To this end, the device exploits photometric stereo to capture the surface properties. The advantage of this technique is that it does not require multiple sensors but only additional lighting that can be arranged compactly. PS allows us to seamlessly recover different surface properties. These properties are then used to describe the SVBRDF of the material. This device, which costs about EUR 80 (about USD 89), is validated by quantitative and qualitative evaluations. Finally, the device is used to acquire multiple materials and build a dataset of textiles to be used in a mixed reality catalog application.

Our device has a wide range of applications across various fields. In digital content creation, it can be used to capture and replicate realistic textures for 3D modeling, gaming, and virtual reality environments. In manufacturing and quality control, it enables precise color and texture matching to ensure consistency and defects detection in products such as fabrics, metals, plastics, and paints. It can be used for visual digital twin creation where the material can be faithfully reproduced, and its appearance can be evaluated with respect to other materials. In e-commerce, it can enhance online shopping experiences by providing accurate visual representations of products in virtual catalogs. The device can also aid in cultural preservation by documenting the appearance of artifacts and historical materials. Furthermore, it has potential uses in scientific research, such as studying surface properties of materials, and in medical fields, where it can assist in analyzing skin textures or other biological surfaces for diagnostic purposes.

The main contributions of this work are, therefore,

- Affordability and Accessibility: The entire system costs are low, as it is entirely built utilizing consumer-grade components in order to democratize material acquisition for both experts and non-experts.
- Streamlined Hardware Design: The device integrates an RGB camera with LED-based lighting in a compact and portable setup, eliminating the need for expensive or bulky equipment.
- Custom Processing Pipeline: We introduce a tailored software pipeline that combines photometric stereo with optimized algorithms to estimate albedo, surface normals, and roughness maps accurately.
- Real-World Applicability: We validate the device’s practicality through its application in creating a mixed reality textile catalog.

The paper is organized as follows: Section 2 reviews the existing literature on material appearance acquisition. Section 3 presents the design and methods of the proposed material appearance acquisition device. In Section 4, we evaluate the accuracy of the device. Section 5 shows an example of the use of our device in a real application. As a use case, we have designed a virtual textile catalog application. Finally, Section 6 concludes the paper with some known limitations and possible solutions for future work.

## 2. Related Work

The existing literature covers the task of material appearance acquisition for both devices and methods. Nowadays, traditional approaches such as the gonioreflectometer are being integrated with newer deep learning techniques. This section describes the most relevant approaches and devices for material appearance acquisition.

### 2.1. BRDF Acquisition

Various devices and methods have been proposed to acquire the BRDF of a material. These methods range from gonioreflectometers [2,5,6] to image-based [3,7,8] and catadioptric [9,10] measurement systems. Here, we are instead interested in obtaining spatial information about the material; thus, we focus on SVBRDF acquisition techniques. Setups for SVBRDF acquisition can be categorized into (hemi-)spherical gantry, photometric stereo, LCD light source, flashlight, and others based on the hardware used for acquisition.

Spherical and hemispherical gantries include the work of Rump et al. [11]. They used a hemispherical gantry with 151 cameras with the cameras’ flashes as light sources. Each camera took one image for each flash (total of 151 × 151 = 22,801 pictures). The images were then processed to produce the material representation of the object. Ghosh et al. [12] proposed three different setups to estimate SVBRDFs of isotropic and anisotropic materials, using up to nine polarized second-order spherical gradient illumination patterns.

In the category of LCD light source systems, Francken et al. [13] and Aittala et al. [14] proposed similar systems based on the use of LCD and an SLR camera. While the recovery process of Francken et al. is limited to the normal map, Aittala et al. can provide SVBRDFs of isotropic surfaces through Bayesian inference. Similarly, Wang et al. [15] used a camera and an LCD screen as an area light source to measure specular and diffuse albedos, two surface roughness parameters, and a 1D power spectrum over frequencies for visible surface bumps. Riviere et al. [16] proposed a mobile reflectometry solution using the LCD of a mobile device as an illumination source in a dimly lit room. Diffuse and specular components were separated by taking two pictures of the same sample with a differently oriented linear polarizer in front of the camera. Albedo, normal, and specular roughness were estimated using the same light patterns described in [12].

The final category of flash illumination setups includes the work of Aittala et al. [17]. A single mobile device is used with its onboard flash to acquire a flash + no-flash image pair of textured material. A multi-stage reconstruction pipeline allows capturing the full anisotropic SVBRDF of surfaces with repetitive patterns. This allows us to assume that multiple points on the surface share the same reflectance properties. The input images are registered by homography, and the image is divided into sub-tiles that roughly match the size of the repeating texture pattern. A master tile is used to compute initial geometric and photometric data of the repeating pattern. This data is then augmented by transferring high-frequency detail from similarly illuminated tiles.

In recent years, deep-learning based methods have emerged to solve the task of material appearance acquisition; these systems usually employ flashlight setup or exploit global lighting. Deschaintre et al. [18] proposed the use of a deep encoder–decoder convolutional neural network to recover per-pixel normal, diffuse albedo, specular albedo, and specular roughness from a single flash-lit picture of a flat surface. They also introduced the rendering loss to support the training process and evaluated the quality of the recovered SVBRDF by comparing its appearance to the ground truth by rendering both under the same lighting configuration. This takes advantage of an in-network render engine that supports inverse-rendering and backpropagation. Similarly, other works [19,20] used encoder–decoder neural networks to estimate the SVBRDF from a single image of a planar surface without needing specular highlights generated by the flashlight. Li et al. [21] use a cascade of encoder–decoder networks to estimate the shape and material appearance of a single image. Gao et al. [22] proposed a deep inverse rendering framework that used [18] and an arbitrary number of images to bootstrap the SVBRDF estimation, which was then refined by a subsequent network to reintroduce fine details lost in the estimation. Deschaintre et al. [23] proposed to use multiple copies of a single image SVBRDF estimation neural network to work with multiple images. The output of each copy of the network was then processed through max pooling and a few convolutional layers to produce the final texture maps.

One of the main challenges and limitations of using deep learning for this task is the necessity of a large amount of training data. While several BRDF datasets are available [24,25,26], SVBRDF datasets with a large number of samples are not available, and these are crucial for the supervised training of deep-learning models. Generating such data manually is a complex and time-consuming task. To solve this problem, Deschaintre et al. [18] used data augmentation over a set of procedural SVBRDFs that were sampled and rendered under multiple lighting directions. Another approach [19,20] used a small training set with known ground truth and a process of self-augmentation to generate additional data for training. Deschaintre et al. [23] further extended their previous approach using an online renderer for data augmentation during training.

### 2.2. Photometric Stereo

One of the techniques that can be used to recover a surface’s properties is photometric stereo (PS). It is a method that uses a similar (but simplified) hardware setup to the hemispherical gantries. The method allows for the recovery of local surface properties from a set of images taken from a single viewpoint illuminated by lights from different directions.

Woodham [4] was the first to introduce PS, proposing an efficient method that exploited the intensity of the captured pixels. In fact, the intensity of each pixel in an image depends on the orientation of the corresponding surface patch (its normal), the reflectance of the material from which the surface is made of, and the direction and spectrum of the lighting. Although the reflectance properties are intrinsic to a surface, its relief produces shades that depend on the direction of the incident illumination. Changes in the illumination direction directly translate into changes in the appearance of a 3D surface. PS exploits this knowledge by using several identical light sources with different placements.

After the initial work of Woodham, several works were presented in the literature to exploit, extend, and adapt the concept of PS for specific applications. Most of the works took advantage of the PS technique to recover the 3D surface variations by using the estimated normal map to generate a depth map. Barsky and Petrou [27], and later Plata et al. [28], proposed the extension of the PS approach to use four light sources instead of three and introduced the use of color images (color PS). Previous works mainly addressed the problem of surface orientation recovery using grayscale images and were not interested in the recovery of color albedo maps. Plata et al. instead used an RGB camera, a single light source, and a turntable to acquire RGB images and recover both the surface orientations and the RGB albedo. Xie et al. [29] proposed to use a PS setup with near point light sources to acquire normal maps of 3D objects with a strong difference in surface orientations. The use of near point lighting creates a nonlinear problem, as the local surface normals are coupled with their distance from the camera as well as from the light sources. They proposed a local/global mesh deformation approach to simultaneously determine the position and the orientation of a facet simultaneously, with each facet corresponding to a pixel in the image. Logothetis et al. [30] used a semi-calibrated near-field PS approach to perform 3D reconstruction. They relaxed the point light source assumption by requiring only known positions rather than intensities; light attenuation maps were computed explicitly. The method can jointly estimate depth, light source brightness, (scaled) albedo, light attenuation maps, and reflectance coefficients. Liu et al. [31] presented a near-light PS algorithm with circularly placed point light sources and a perspective camera. In their work, they modeled the captured scene as a 3D triangulated mesh whose vertices corresponded to the observed pixels. They used a two-stage process to first solve PS using the differential images captured by changing the light source position by a small amount along a circular path, and later refined the vertex positions using the original image formation model applied to the raw captured images. Their algorithm is sensitive to calibration errors, so they proposed an accurate light source position estimation approach using a flat panel display. Li et al. [32] presented a method to capture both the 3D shape and spatially varying reflectance of isotropic materials using a multi-view PS. They combined the structure from motion technique [33] to obtain precise 3D reconstruction and capture the SVBRDF by simultaneously inferring a set of basis BRDFs and their mixing weights at each surface point.

All of the above-mentioned works use complex hardware setups that require expensive specialized hardware, such as industrial equipment or laboratory prototypes. However, a few portable PS-based devices have been proposed in the literature. Gorpas et al. [34] proposed a miniature PS system aimed at three-dimensional structural reconstruction of various types of fabrics. Their goal was to develop a robotic system that could navigate in unstructured environments, identify and retrieve textiles from piles or containers, and untangle and spread the textiles for some industrial process or folding. Their system consisted of a low-cost, off-the-shelf camera operating in macro mode and eight light-emitting diodes (LEDs). The device was a cylinder of about 30 mm in diameter and height. It was able to acquire a 10 × 10 mm textile patch with a resolution of 400 × 400 pixels. Only grayscale images were used to acquire 3D geometry and albedo maps. Kampouris et al. [35] built a small portable device to acquire a 10 × 10 mm patch of textiles to capture the microstructure of the fabrics. Their system used an RGB camera and four LED light sources to acquire a normal map and grayscale albedo of the textiles. They used this recovered information to address the problem of textile classification using both handcrafted and deep-learning features using normal maps and albedo instead of plain images. Finally, Schmitt et al. [36] proposed a handheld device for the joint estimation of the pose, geometry, and SVBRDF. While the device was portable, it used a complex hardware design and software pipeline. The device used a Kinect-like active depth sensor, a global shutter RGB camera, and 12 point light sources (high-power LEDs) surrounding the camera in two circles (with radii of 10 cm and 25 cm). They estimated the pose using structure from motion and created a volumetric representation of the object by fusing the acquired depth maps. Normals and albedo were initialized assuming Lambertian reflectance. Specular BRDF parameters were initialized as a uniform mixture of base materials and were later refined by optimizing (gradient-based optimization) their combination, minimizing the photometric error.

Table 1 compares the main characteristics of the most relevant approaches and devices for material appearance acquisition.

## 3. The Proposed SVBRDF Acquisition Device

This section describes the proposed SVBRDF acquisition device to solve the problem of material appearance acquisition. Due to the wide variety of real-world materials, the scope of the device is restricted, and some assumptions are made before describing the hardware and the software pipeline for the acquisition.

### 3.1. Scope and Design Choices

The goal of the proposed device is to be able to acquire the SVBRDF of a patch of a surface. Some constraints of the surface and its material are applied.

The device must be portable and usable by people who are not experts in the field. Aiming for widespread use of the device, its cost should also be limited. The materials acquired must be of a planar surface with micro surface height variations in the order of a few millimeters. While building a device for capturing the SVBRDF of generic materials and shapes is possible, it complicates the design of the hardware and makes it difficult to build a portable device. In addition to this, huge variations in the micro surface also need 3D geometry to be considered. Some restriction on the kind of materials also applies. At present, only opaque materials are taken into consideration. Transparent, translucent, as well as highly specular materials (e.g., mirrors, metals) are excluded. The patch to be taken into consideration should be of at least of size 5×5 cm; this allows for meaningful variations in the characteristics of the material to be included while keeping the device size limited, thus making it a portable device. This size also allows for the reproduction of the materials on displays (e.g., smartphones) matching the screen size to the real size of the acquired material patch.

Out of all of the techniques used for material appearance acquisition, our device is built upon photometric stereo, which was chosen for several reasons. It allows for building a device that is compact and made of inexpensive consumer components. It is also a static device with no moving parts that could be damaged during the transport of the device. For example, a gonioreflectometer-like or gantry setup has high hardware costs and is not suitable for a mobile device. LCD-based devices are often cheaper, but they are still not suitable for building turnkey portable devices. Statistical-based methods rely heavily on dense pattern repetition to provide accurate acquisitions, and this may not be true when capturing a small patch of the surface. Deep learning-based methods are interesting because they can provide materials’ BRDF using RGB images and do not require specialized hardware; however, they require huge computing capabilities for both training and inference. They also require a large amount of data for the training process, and such data are not easily collected or publicly available in large quantities. Like many of the other techniques, photometric stereo requires some calibration, but since the device is self-contained, this can be achieved during production rather than by the user.

The developed device uses the SVBRDF extension of the following BRDF. The Cook–Torrance BRDF [38] is used as the base reflectance representation, but a few changes are made. First, Shlick’s approximation of the Fresnel term is adopted. Both the microfacet distribution and the shadowing and masking terms of the Cook–Torrance model are replaced by the GGX formulation proposed by [39]. This BRDF was chosen among the others since it fits the classes of materials that the device aims to capture; it is also a common formulation of the BRDF adopted by many rendering engines. By providing the material appearance in the same format, it is possible to render it in the engines without having to convert between different representations or define new shaders in the engines.

### 3.2. Hardware

For the photometric stereo approach, some essential components are needed: a digital camera, a variable number of light sources, and a setup that allows acquisition without the interference of external light sources.

We aim at an affordable hardware; thus, the device builds on the most suitable cheap electronics available on the consumer market and uses a custom case to house all of the components. Specifically, the device uses the Raspberry Pi Camera Module V2, SK6812 LEDs and the Raspberry Pi Zero W. (Raspberry Pi Ltd., Cambridge, UK). The complete bill for the materials and the price of each component are reported in Table 2.

#### 3.2.1. Digital Camera Module

Out of all the available camera modules, the Raspberry Pi Camera Module V2, https://www.raspberrypi.com/documentation/accessories/camera.html, (accessed on 10 February 2025) was chosen. It is a camera sensor module that comes in the form of a small (25×24 mm) breakout board mounted with a Sony IMX219 (Raspberry Pi Ltd., Cambridge, UK) [40] CMOS image sensor. This mobile-format sensor (3.68×2.76 mm, pixel size 1.12 µm) provides 8 MP resolution color images (3280×2464 px) and is equipped with a small adjustable lens with a focal length of 3.04 mm (62.2° horizontal field of view). The choice of this specific module was based on three different factors: First, the camera satisfies the image acquisition requirements; it can acquire an area of size 82×61 mm with a spatial resolution of 40 px/mm when placed 68 mm above the target surface. Second, the camera module is provided with libraries that allow for the acquisition of images using a pre-built camera pipeline as well as RAW-10 images. The ability to acquire RAW images is essential for building a custom camera pipeline for the device. Finally, the module is inexpensive (market price of USD 25) and easily interfaces with the Raspberry Pi Single Board Computers (SBCs) as well as third-party SBCs thanks to its camera serial interface (CSI).

#### 3.2.2. Light Sources

In order for the device to work properly, it is essential to be able to illuminate the surface with different light source placements. While several types of light sources are available on the market, the light emitting diode (LED) is known for its efficiency and the large number of variations (shape, power, and wavelength). The most important factors in selecting LEDs are the wavelength of the emitted light, ease of wiring, mechanical assembly, power consumption, and emission.

The selected light sources are thus pre-built rings mounting SK6812 RGBW LEDs [41]. The SK6812 LED is a 5050 LED chip (5×5 mm) that comes in a few different variations; the most suitable for this application is the RGB + Cold White. The cold white variation has been chosen since it provides a white color temperature of approximately 6500 K, which is equal to the CIE standard illuminant D65 which resembles the daylight illuminant. The white channel emits 6±1 lumens, and the light beam angle is 120°. This LED also offers some advantages over other LEDs in terms of light control and wiring. The wiring diagram consists of a 5 V power supply to be provided to each LED and a single wire control bus to daisy-chain the LEDs. Since the LEDs are already provided and assembled on PCB rings, it is only necessary to wire the power supply and the control bus to the first LED of each ring. Furthermore, inexpensive pre-assembled LED rings are available in diameters ranging from 32 mm (8 LEDs) up to 112 mm (32 LEDs).

Two rings were used in the device, with 8 and 16 LEDs, respectively, for a total of 24 light sources. The choice was based on mechanical constraints; the rings should not interfere with the camera’s field of view, and each LED must provide enough energy to each point in the area portrayed by the camera. Moreover, the use of a large number of light sources helps deal with noise and shadows in the acquired images. Exact LEDs placement is described later in this section.

#### 3.2.3. Controller Board

The final electronic component required is a controller board, which is responsible for controlling the image acquisition process. Two different approaches can be employed. The first one is to use this controller board only for the acquisition and delegate the processing to software running on a computer. The second approach involves using the controller not only for acquisition but also for image processing. The second case requires more computational capabilities. Since a computer is, in any case, needed to recover the texture maps, the device employs the first solution to also keep the hardware of the device limited in size, power consumption, and cost. Based on this reasoning and the hardware already selected for the camera module and the light sources, the Raspberry Pi Zero W, https://www.raspberrypi.com/products/raspberry-pi-zero-w/, (accessed on 10 February 2025) is the designated SBC to control the images acquisition process. It has a 1 GHz single-core CPU, 512 MB of RAM, a CSI camera interface, a wireless network adapter, and a general-purpose input/output (GPIO) interface to control the LEDs.

Power is supplied via the Raspberry’s micro-USB power connector. Since the power consumption of the LEDs does not exceed the recommended maximum of 1 A for the 5 V rail, no external voltage regulator is required. A single GPIO pin is used to drive all of the LEDs; refer to the SK6812 datasheet [41] for details on the control protocol. A Wi-Fi connection is used for communication and data transfer between the device and the computer.

#### 3.2.4. Device Case

**Figure 1:**
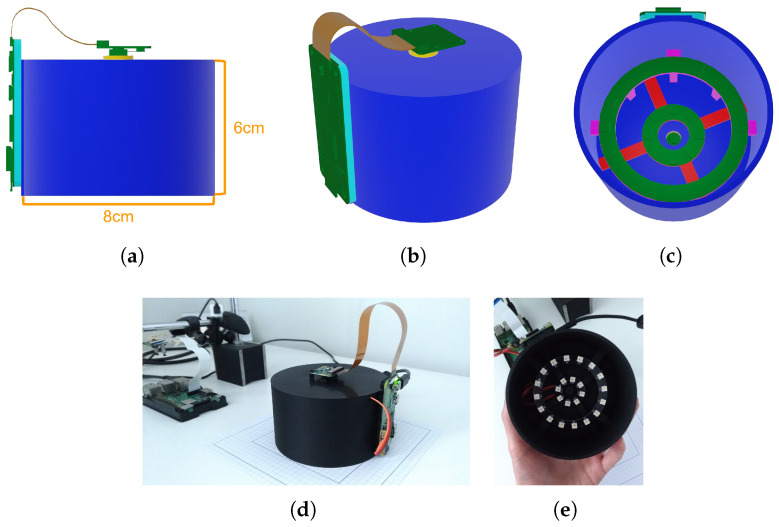
CAD drawings of the device (**a**–**c**) and the assembled device (**d**,**e**).

A 3D-printed plastic case was designed to hold the hardware in place and block the light coming from external sources, thus providing a dark room for the material appearance acquisition. Figure 1 shows computer-aided design (CAD) sketches and the final device. Since the housing was 3D printed using PLA, which is highly specular, the inner surface of the device was painted with a matte black color to limit the effects of reflections from the case walls in the acquired images.

The exact placement of the camera and LED rings has been determined to guarantee that a patch of material (5×5 cm in size) is acquired, with each point on the surface receiving enough light for each LED. Therefore, the camera is placed 68 mm above the surface. The two LED rings are instead placed as follows: the smaller one, which has a radius of 13 mm (center of the ring to center of the LED), is located 45 mm above the surface, forming a 75° angle with the surface. The larger one (33 mm radius) is instead positioned 40 mm above the surface, producing a 51° incident light angle. Both rings are aligned so that their center corresponds to the main view axis of the camera.

### 3.3. Software

The material appearance acquisition software consists of two main modules: a firmware that runs on the Raspberry Pi Zero to control the LEDs and image acquisition and a processing pipeline that runs on a personal computer.

#### 3.3.1. Onboard Firmware

Due to the limited computational power and RAM, the goal of the onboard firmware is to coordinate the image acquisition process and transfer the data to the personal computer where the processing takes place. The onboard firmware accepts commands from the main elaboration pipeline and mainly controls the device’s LEDs and camera. This software runs on the Raspberry Pi Zero, and the commands and data are exchanged through an HTTP API using the Flask framework. The Raspberry Camera module is controlled through a customized version of the Picamera library, https://github.com/waveform80/picamera, (accessed on 10 February 2025). The LEDs are driven using the Adafruit CircuitPython NeoPixel library, https://github.com/adafruit/Adafruit_CircuitPython_NeoPixel, (accessed on 10 February 2025). The image transmission uses binary streams, while supplementary data are transferred using JavaScript object notation (JSON) documents.

#### 3.3.2. Main Software and Processing Pipeline

**Figure 2:**
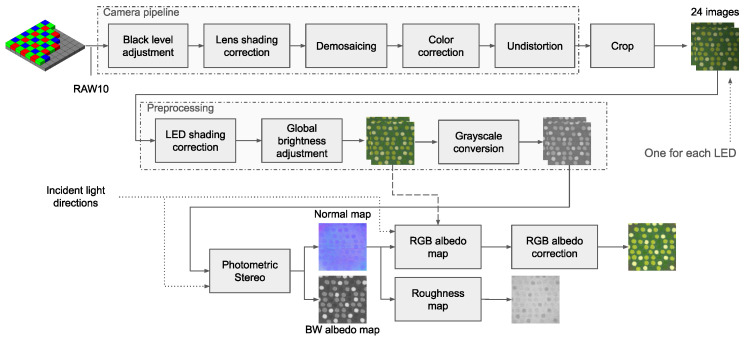
Main software pipeline.

The software pipeline consists of three main blocks: the camera pipeline, the preprocessing, and the photometric stereo processing. The camera pipeline takes as input the RAW Bayer pattern data from the camera sensor and converts this information into an RGB image. A total of 24 images are acquired for each sample, one image for each LED. The preprocessing applies a series of operations (see Figure 2) to ensure consistency between the acquired images; LED shading correction and global brightness adjustment are the most important. Finally, photometric stereo estimates the material appearance from the images and produces the normal map, albedo map, and roughness map.

In more detail, the camera pipeline performs the following steps: black level adjustment, lens shading correction, demosaicing, color correction, and lens undistortion. Some of these steps require calibration; see Section 3.4 for details. Black level adjustment is performed according to the camera sensor datasheet. Lens shading correction is applied using a pixel-wise multiplicative calibrated correction matrix. The demosaicing step uses the weighted average demosaicing approach to convert the RAW image to an RGB image. Color correction is then applied to account for sensor nonlinearity ($a\cdot RGB^{2}+b\cdot RGB+c$), RGB correction (M·RGB, where M is a 3×3 linear color correction matrix), and gamma correction ($RGB^{\gamma }$). Next, the lens radial distortion is corrected. Finally, the image is cropped to a square matching the 5 × 5 cm patch of the acquired tile (approximately 2048×2048 pixels). The camera pipeline is used to acquire 24 images, using the same camera acquisition setup but a different LED for each of image.

Some image pre-processing is then applied to ensure consistency between the 24 images. LED shading correction takes into account the uneven light energy across the surface. Since the distance from the LED differs for each point on the surface and the emission lobe of the LED is not a perfect hemisphere, the more distant part of the surface is usually less illuminated than the part directly under the light source. This step corrects the image so that the entire surface has the same brightness. The correction is conducted using a multiplicative matrix, similar to the lens vignetting correction. Then, the global brightness of the remaining 23 images is adjusted to match the average global brightness of the first image. This takes into account tolerances in the power emitted by the different LEDs. The final pre-processing step creates the grayscale copies of the RGB images that will be used in the PS pipeline.

After pre-processing PS is applied to recover the albedo and normal maps. The PS equation assumes that the incoming light has the same incident direction for each point on the surface. This is usually true if the light source is a point light placed far from the surface. In this device, light sources are LEDs placed close to the acquired surface and can be approximated to quasi-point light sources. As such, the incident light direction is different for each point. To account for this, the shading function of the PS equation is rewritten as in Equation (1), which defines I(x,y) the intensity of each pixel of coordinates x,y in the image.

(1) $$I\left(x,y\right)=\rho \left(x,y\right)L_{i}L\left(x,y\right)\cdot N\left(x,y\right)$$

ρ(x,y) defines the albedo, and $L_{i}$ is the intensity of the incident light. The term L(x,y) defines the incident light direction and uses a different direction vector for each pixel in the image. Finally, N(x,y) is the surface normal vector. Thanks to the LED shading correction and global brightness adjustment steps, it can be assumed that the incident light intensity is equal for each pixel and for each LED, so $L_{i}=1$. Equation (1) can be rewritten for each pixel as Equation (2).

(2) $$\begin{array}{cc} I & =\rho L\cdot N=\rho N^{T}L\\ I & =G^{T}L=L^{T}G,\;\mathrm{where}\;G=\rho N \end{array}$$

Considering all of the 24 equations (one per image) it is possible to solve the system of Equation (3) for G and obtain a vector for each pixel that encodes the normal direction *N* and the grayscale albedo ρ (see Equation (4)).

(3) $$\left\{\begin{array}{cc} I_{1} & =L_{1}^{T}G\\ \vdots \\ I_{24} & =L_{24}^{T}G \end{array}\right.$$

(4) $$N=\frac{G}{\left||G|\right|},\;\rho =\left||G|\right|$$

To obtain the color albedo, Equation (5) is minimized for each RGB channel separately. This is achieved by computing the value of Equation (6).

(5) $$Q\left(\rho \right)=\sum_{i=1}^{24}{\left(I_{i}-\rho L_{i}^{T}N\right)}^{2}$$

(6) $$\begin{array}{cc} \frac{dQ(\rho )}{d\rho } & =-2\sum_{i=1}^{24}\left(I_{i}-\rho L_{i}^{T}N\right)L_{i}^{T}N\\ \frac{dQ(\rho )}{d\rho } & =0\Rightarrow \rho =\frac{\sum_{i=1}^{24}\left(I_{i}L_{i}^{T}N\right)}{\sum_{i=1}^{24}\left(L_{i}^{T}N\right)} \end{array}$$

**Figure 3:**
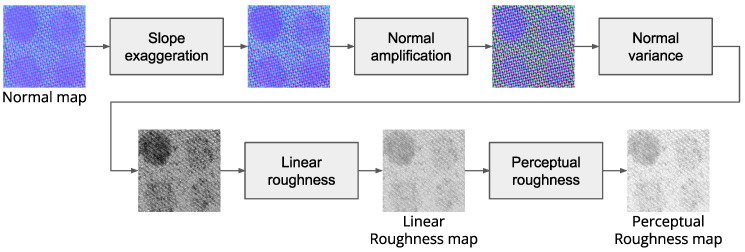
Roughness estimation pipeline.

After obtaining the normal and albedo maps, it is possible to recover the roughness map. According to [42], the variance of the normal map can be used as an indicator of the surface roughness. Following their intuition, here, a pipeline is defined that can provide an estimation of the roughness map from the normal map recovered through the PS. The steps of the pipelines are visible in Figure 3. Consider *n* as the estimated normal map where for each pixel the surface normal is defined as a vector with three components $\left[n_{x},n_{y},n_{z}\right]$. The first operation is the slope exaggeration (Equation (7)) which allows for the small surface normal perturbations to be mapped into larger changes by exaggerating the component of the normal that points away from the vertical axis. Hereafter, normals are amplified using a high-frequency emphasis filter for surface normal (Equation (8)) that considers a window around each pixel of the image. Those two steps are meant to boost the variability of the normal directions in preparation for the variance computation. Constant values *s*, *m*, and *k* where empirically found through experimentation with different materials and are constrained to the hardware setup of the device. The variance $\sigma^{2}$ is later computed through Equation (9) which considers a window around each pixel.

(7) $$n^{*}=\frac{n_{s}={\left(n_{x},n_{y},n_{z}/s\right)}^{T}}{\left|\right|n_{s}\left|\right|},\;s=1.5$$

(8) $$n^{\prime }=n^{*}+k\left(n^{*}-\frac{n_{a}=\sum_{j=1}^{m}n_{j}^{*}}{\left|\right|n_{a}\left|\right|}\right),\;m=23\times 23\;\mathrm{px},k=2.5$$

(9) $$\sigma^{2}=\frac{\sum_{j=1}^{m}\left({\left({n^{\prime }}_{jx}-{\bar{n^{\prime }}}_{x}\right)}^{2}+{\left({n^{\prime }}_{jy}-{\bar{n^{\prime }}}_{y}\right)}^{2}+{\left({n^{\prime }}_{jz}-{\bar{n^{\prime }}}_{z}\right)}^{2}\right)}{m},\;m=23\times 23\;\mathrm{px}$$

The linear roughness value *r* is then computed using Equation (10) to better fit the 0–1 range.

(10) $$r=\sigma^{2}k_{2},\;k_{2}=150$$

Finally, the perceptual roughness as leveraged by rendering engines is obtained by computing the square root of the linear roughness ($r_{p}=\sqrt{r}$).

The average execution time for material acquisition is 6 min 00 s on an Intel Core i7 6700K processor. Specifically, image acquisition and data transfer from the device to the PC takes 2 min 35 s, and pipeline execution takes the remaining 3 min 25 s. It should be noted that the current code does not exploit any optimization or high performance computing capabilities.

### 3.4. Device Calibration

A few calibration procedures are required to build the calibration data used for some of the main pipeline steps.

#### 3.4.1. Lens Shading Correction Calibration

**Figure 4:**
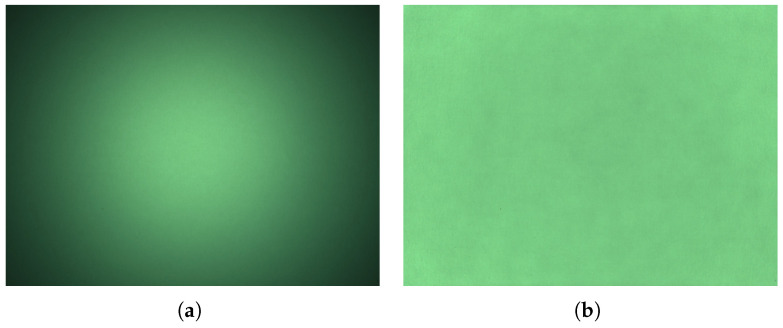
RAW image before (**a**) and after (**b**) lens shading correction.

In this step, we estimate a set of parameters to correct for the vignetting introduced into the image by the lens. The correction factors were computed by using a RAW Bayer image of a completely white tablet screen. To obtain such an image, a translucent white sheet of paper was placed directly over the screen since the ground sample distance (GSD) of the camera was small enough to distinguish the color filters of the LCD screen. Figure 4 shows an example of a RAW image before and after lens shading correction. The channels of the captured RAW image are separated based on the *rgGb* Bayer pattern. For each channel, the center 64×64 px window is used to define the desired average brightness level ${avg}_{ch}$; average blur is then used to clear noise. The correction factors are computed as ${avg}_{ch}/{image}_{ch}$.

#### 3.4.2. Color Correction Calibration

This calibration estimates the parameters a, b, c, M, and γ used to correct the camera’s sensor nonlinearity, RGB color, and gamma encoding. The parameters are optimized using the Nelder–Mead simplex [43] method over color data acquired from an X-Rite ColorChecker Classic Mini. Due to the size of the color checker, each square was captured separately but using the same camera parameters.

#### 3.4.3. Lens Undistortion Calibration

Due to the shallow depth of field and mechanical mount of the camera, the lens radial distortion parameters must be manually calibrated. Parameters for the Brown distortion model [44] were manually estimated using a black-and-white checker board pattern.

#### 3.4.4. LED Shading Correction Calibration

Similar to lens shading correction, this is the process of estimating the correction of the shading introduced in the image by the LEDs. One of the core assumptions of PS is that the energy that lights up the material is equal for each point on the surface. When using light sources close to the surface, this is not true, and light fall-off can be observed across the images. To compensate for the light fall-off, a correction matrix was computed for each of the 24 LEDs by taking an image of a uniform white paper sheet. The acquired image is then cleared of noise using average blur. Correction factors are then computed for each RGB channel separately.

#### 3.4.5. Incident Light Direction Calibration

**Figure 5:**
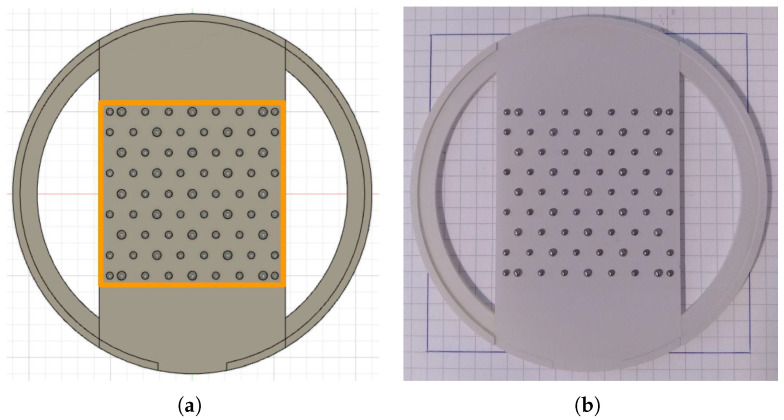
Incident light direction calibration target. (**a**) CAD drawing, in orange the camera field of view. (**b**) 3D-printed target.

Given that the light sources are not point light placed at infinity, but quasi-point light placed near the surface to be acquired, the incident light direction depends on the LED placement, and it also varies along the surface. To provide the correct light direction vector for each pixel acquired by the camera, a physical calibration target with 71 mirror-like spheres was designed (see Figure 5). The spheres are arranged in a hexagonal pattern to allow for later interpolation. The target structure was printed using a resin-based 3D printer with an XY resolution of 47 μm and Z (layer height) of 10 μm.

**Figure 6:**
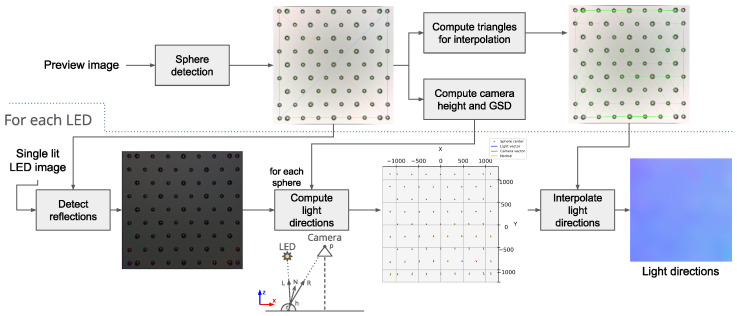
Incident light direction calibration pipeline.

The calibration pipeline executes the steps visible in Figure 6. An image is acquired using all the LEDs to detect positions and contours of the spheres. The spheres’ centers are also used to determine the Delaunay tessellation later used for interpolation of the light directions, the boundary of the usable region-of-interest, and the length in pixels of the diagonals of the calibration target. Knowing the real length of the target’s diagonals and the camera’s intrinsic parameters, it is now possible to compute the exact height of the camera from the surface.

An image for each LED is acquired and then processed through various steps. First, the reflections over the spheres are detected by finding the brightest spots. Then, the light direction is computed using the equation L=2(N·R)N−R, where N is the normal direction at the brightest spot on the sphere, and R is the vector of the direction reaching the camera through the focal plane. The 71 known light direction vectors are then used to build the full incident light vector map by using barycentric interpolation.

#### 3.4.6. Albedo Correction Calibration

This calibration uses the Finlayson 2015 color correction method [45] to estimate the color correction to be applied to the estimated color albedo map. The correction parameters are estimated using a GretagMacbeth ColorChecker DC color chart. This chart consists of 237 color patches. The eight glossy patches are excluded, and repeated patches are considered only once. Since the chart exceeds the acquisition size of the device, the tiles are acquired and their albedo is estimated in groups of 4 (2 by 2 patches) to speed up the process. The same camera parameters are used for all of the acquisitions.

## 4. Device Accuracy Evaluation

A common approach to evaluate PS-based methods is to use synthetic images for which the ground truth of the SVBRDF maps is known. This approach works well for assessing modifications to the PS pipeline, but it is not sufficient in our case. In addition to PS, it is necessary to evaluate the hardware assembly and its calibration as well as the camera pipeline and the pre-/post-processing steps. Therefore, a procedure is defined for evaluating the device as a whole, enabling evaluation of the various aspects involved in the material appearance acquisition.

### 4.1. Evaluation Target

To evaluate the quality of the computed normal and albedo maps of the surface, a physical target with known variations in normal direction and uniform color was designed.

**Figure 7:**
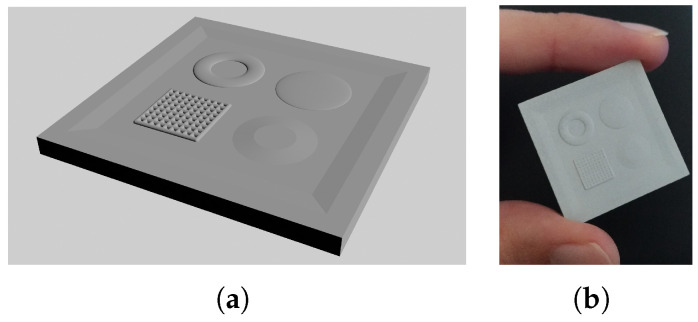
Evaluation target. (**a**) CAD drawing. (**b**) 3D-printed target.

This tool is a square of size 30×30×2 mm and presents four different shapes with a height variation of 0.25 mm (see Figure 7a). In detail, following the clockwise rotation from the top left, we have a torus to stress the normal map at high slant angles, a flattened hemisphere to test the normal estimation at lower slant angles, a truncated cone to test constant normal angles at different heights, and a grid pattern to simulate the variations of a textile. The first three shapes are also useful to test the variations of the normals for all of the possible rotations around the vertical axis. Finally, the four shapes are placed in an area lowered by 0.25 mm to have their highest portion at the same height as the edges.

The evaluation target was printed using the same resin-based 3D printer already used for the light direction calibration target. The 3D-printed tool was airbrushed with a uniform light gray matte coating. The final result is visible in Figure 7b.

### 4.2. Albedo Map Evaluation

The estimated albedo map of the material is evaluated in terms of both color accuracy and color uniformity. The former evaluates the ability of the device to provide perceptually correct albedo maps, while the latter evaluates its ability to produce correct albedo maps for uniform materials but with varying normal directions.

#### 4.2.1. Color Accuracy

Evaluation of the color accuracy of the estimated albedo map is performed using the GretagMacbeth ColorChecker DC color chart. This uses the same chart and acquisition procedure previously used for albedo correction calibration (see Section 3.4.6).

As pointed out by [46], the estimation and evaluation of the albedo map presents the challenge of collimating the absolute brightness of the estimate with that of the ground truth. The difference in global brightness is due to the acquisition setup and can be easily dealt with when performing later re-renderings; for this reason, the evaluation should not consider mere differences in global brightness. In that work, a scale-invariant MSE metric is defined to evaluate the albedo. The scale invariance is enforced by a scalar α, which is optimized to minimize the error, thus taking into account the ambiguity in the absolute brightness of the scene or absolute intensity of the albedo.

While their proposal works for grayscale albedo, extending this concept to color albedo is not a trivial process. Following their reasoning, a scale-invariant version of the $\Delta E_{00}^{*}$ metric is proposed. The $\Delta E_{00}^{*}$ [47] was defined by the International Commission on Illumination (CIE) as a way to measure the perceived visual difference between two colors in the CIELAB color space. Delta E is a metric for understanding how the human eye perceives color differences. The presented modification uses a scalar α multiplied by the lightness to account for different global brightness between the estimated albedo values and the reference ones. The complete metric is reported in Equation (11).

Since the albedo texture encodes only the base color of the surface with no information about light temperature or intensity, evaluation is performed against the ground truth colors of the ColorChecker as acquired under an ideal E illuminant, which is an equal energy generator that provides a constant SPD in the visible spectrum.

(11) $$\begin{array}{cc} si-\Delta E_{00}^{*} & =\frac{1}{n}\min_{\alpha }\sum \left(\sqrt{{\left(\frac{\Delta L^{\prime }}{k_{L}S_{L}}\right)}^{2}+{\left(\frac{\Delta C^{\prime }}{k_{C}S_{C}}\right)}^{2}+{\left(\frac{\Delta H^{\prime }}{k_{H}S_{H}}\right)}^{2}+R_{T}\frac{\Delta C^{\prime }}{k_{C}S_{C}}\frac{\Delta H^{\prime }}{k_{H}S_{H}}}\right)\\ \Delta L^{\prime } & =L_{2}^{*}-L_{1}^{*}\alpha \;k_{L},\;k_{C},\;k_{H}\;\mathrm{default}\;1\\ {\bar{L}}^{\prime } & =\frac{L_{1}^{*}\alpha +L_{2}^{*}}{2}\;\bar{C}=\frac{C_{1}^{*}+C_{2}^{*}}{2}\;C_{1}^{*}=\sqrt{{\left(a_{1}^{*}\right)}^{2}+{\left(b_{1}^{*}\right)}^{2}}\;C_{2}^{*}=\sqrt{{\left(a_{2}^{*}\right)}^{2}+{\left(b_{2}^{*}\right)}^{2}}\\ a_{1}^{\prime } & =a_{1}^{*}+\frac{a_{1}^{*}}{2}\left(1-\sqrt{\frac{{\bar{C}}^{7}}{{\bar{C}}^{7}+25^{7}}}\right)\;a_{2}^{\prime }=a_{2}^{*}+\frac{a_{2}^{*}}{2}\left(1-\sqrt{\frac{{\bar{C}}^{7}}{{\bar{C}}^{7}+25^{7}}}\right)\\ C_{1}^{\prime } & =\sqrt{{a_{1}^{\prime }}^{2}+{b_{1}^{*}}^{2}}\;C_{2}^{\prime }=\sqrt{{a_{2}^{\prime }}^{2}+{b_{2}^{*}}^{2}}\\ {\bar{C}}^{\prime } & =\frac{C_{1}^{\prime }+C_{2}^{\prime }}{2}\\ \Delta C^{\prime } & =C_{2}^{\prime }-C_{1}^{\prime }\\ h_{1}^{\prime } & =atan2\left(b_{1}^{*},a_{1}^{\prime }\right)\;\;\mathrm{mod}\;\;360{}^{\circ}\;h_{2}^{\prime }=atan2\left(b_{2}^{*},a_{2}^{\prime }\right)\;\;\mathrm{mod}\;\;360{}^{\circ}\\ \Delta h^{\prime } & =\left\{\begin{array}{cc} h_{2}^{\prime }-h_{1}^{\prime } & \mathrm{if}\;|h_{1}^{\prime }-h_{2}^{\prime }|\;\leq 180{}^{\circ}\\ h_{2}^{\prime }-h_{1}^{\prime }+360{}^{\circ} & \mathrm{if}\;|h_{1}^{\prime }-h_{2}^{\prime }|\;>180{}^{\circ},h_{2}^{\prime }\leq h_{1}^{\prime }\\ h_{2}^{\prime }-h_{1}^{\prime }-360{}^{\circ} & \mathrm{if}\;|h_{1}^{\prime }-h_{2}^{\prime }|\;>180{}^{\circ},h_{2}^{\prime }>h_{1}^{\prime } \end{array}\right.\\ \Delta H^{\prime } & =2\sqrt{C_{1}^{\prime }C_{2}^{\prime }}\sin\left(\frac{\Delta h^{\prime }}{2}\right)\\ {\bar{H}}^{\prime } & =\left\{\begin{array}{cc} \left(h_{1}^{\prime }+h_{2}^{\prime }+360{}^{\circ}\right)/2 & \mathrm{if}\;|h_{1}^{\prime }-h_{2}^{\prime }|\;>180{}^{\circ}\\ \left(h_{1}^{\prime }+h_{2}^{\prime }\right)/2 & \mathrm{if}\;|h_{1}^{\prime }-h_{2}^{\prime }|\;\leq 180{}^{\circ} \end{array}\right.\\ T & =1-0.17\cos\left({\bar{H}}^{\prime }-30{}^{\circ}\right)+0.24\cos\left(2{\bar{H}}^{\prime }\right)+0.32\cos\left(3{\bar{H}}^{\prime }+6{}^{\circ}\right)-0.2\cos\left(4{\bar{H}}^{\prime }-63{}^{\circ}\right)\\ S_{L} & =1+\frac{0.015{\left({\bar{L}}^{\prime }-50\right)}^{2}}{\sqrt{20+{\left({\bar{L}}^{\prime }-50\right)}^{2}}}\;S_{C}=1+0.045{\bar{C}}^{\prime }\;S_{H}=1+0.015{\bar{C}}^{\prime }T\\ R_{T} & =-2\sqrt{\frac{{{\bar{C}}^{\prime }}^{7}}{{{\bar{C}}^{\prime }}^{7}+25^{7}}}\sin\left\{60{}^{\circ}\cdot \exp\left[-{\left(\frac{{\bar{H}}^{\prime }-275{}^{\circ}}{25}\right)}^{2}\right]\right\} \end{array}$$

**Figure 8:**
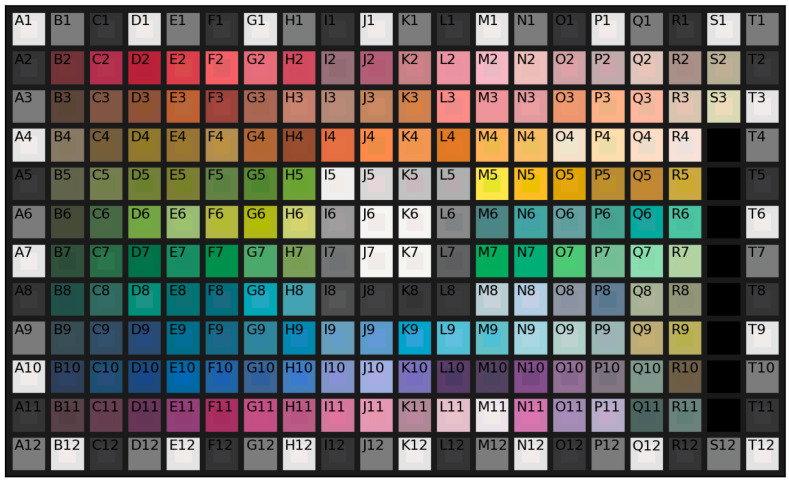
DC ColorChecker comparison between ground truth and estimated albedo. In each square, the inner part is the estimated albedo, while the outer part is the ground truth. (Best viewed in digital format and zoomed in).

Albedo color evaluation is performed using the $si-\Delta E_{00}^{*}$ following the leave-one-out cross-validation procedure on the acquired color chart data. The obtained average $si-\Delta E_{00}^{*}$ is equal to 2.89, indicating a perceptual error that is limited but still observable by the human eye. When estimating the correction parameters using all the available patches simultaneously, the error is about 2.85. Before the albedo color correction, the average $si-\Delta E_{00}^{*}$ is equal to 3.62. Figure 8 shows the comparison between the desired ground truth albedo colors and the obtained albedo after correction. Note that the larger errors are evenly distributed across the different shades.

#### 4.2.2. Color Uniformity

The color uniformity evaluation aims to test the ability of the device to correctly estimate the albedo as the normal direction varies. This evaluation uses the tool introduced in Section 4.1. To perform this evaluation under ideal conditions, the tool used for evaluation should present a Lambertian surface. Since Lambertian surfaces cannot be easily created in the real world, the target uses a uniform matte gray paint that approximates their characteristics.

The evaluation of the color uniformity of the albedo uses the $\Delta C_{00}^{*}$ metric. This metric is based on $\Delta E_{00}^{*}$ but does not consider the lightness channel in the evaluation. Furthermore, since it was not possible to characterize the reflective properties of the painting used, and since this evaluation aims only to measure the uniformity of the albedo, not its colorimetric accuracy (which is evaluated instead in the previous paragraph), the evaluation is made against the average color of the acquired albedo map. The evaluation presents a $\Delta C_{00}^{*}=1.89$, which indicates that the device can correctly acquire the albedo of a material with a limited error even with changes in the normal directions.

### 4.3. Normal Map Evaluation

**Figure 9:**
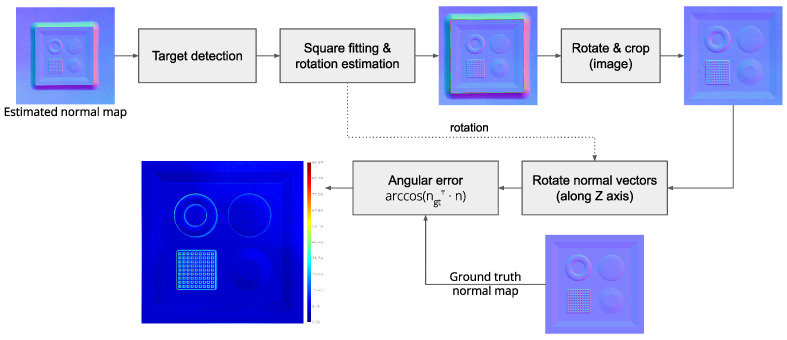
Normal map evaluation pipeline and heatmap.

The quality of the computed normal map is evaluated using the tool described in Section 4.1. The SVBRDF of the target is acquired by running the software pipeline. The obtained normal map is evaluated following the pipeline defined in Figure 9. First, the location of the target is detected by creating a mask using thresholding, flood fill, erode, and dilate operations. Edges of the target are then detected using the Canny edge detector, and a square is fitted on the edges using the algorithm defined by [48]. Once target detection is complete, an affine 2D transformation is estimated and applied to account for the rotation of the target around the vertical axis with respect to the camera. The corrected image is then cropped to contain only pixels belonging to the target. Since the contents of the image are 3D vectors, the same rotation correction applied in the image space is also applied to the vectors with respect to the vertical axis Z. Finally, the angular error in degrees, defined in Equation (12), is computed against the ground truth normal map ($n_{gt}$) of the target.

(12) $$AngularError=\arccos\left(n_{gt}^{⊺}\cdot n\right)$$

The result of the normal map evaluation shows a mean angular error of 9.5° and a median of 7.42° (min = 0.0°, max = 96.98°, 1st quartile = 7.27°, 3rd quartile = 10.40°). By taking a closer look at the error heatmap in Figure 9, it is clear that the error is higher around the edges of the torus and the grid pattern where the slant is greater.

### 4.4. Roughness Map Evaluation

To the best of our knowledge, no method for quantitative evaluation of the quality of roughness texture maps of the real surface has been defined in the literature. Existing evaluation methods are thought to quantize the roughness of real surfaces for mechanical analysis purposes. While this is useful for some tasks, it is not sufficient to evaluate a roughness map since it encodes different information.

Metrics such as $R_{a}$ [49] measure physical properties such as the arithmetic mean roughness value of the profile deviations from the mean line of the roughness as a single real number for the entire surface. Instead, the roughness texture map describes the micro-facet distribution of each pixel separately.

**Figure 10:**
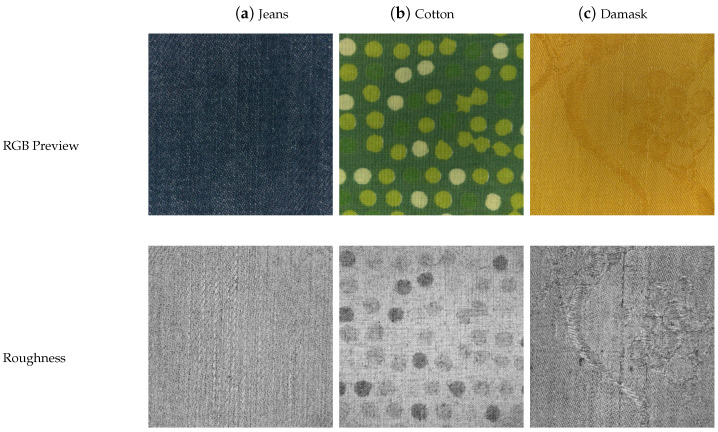
Samples of acquired roughness maps.

The main limitation of the current process of roughness map estimation is that it uses various experimentally found values (i.e., window size and scaling factors). While fixed values provide acceptable results, adjustments to those values may be needed for some kinds of materials. In general, the pipeline tends to provide too glossy roughness, but the problem is partly mitigated by the presence of the normal map. This is partly due to the map being derived for each pixel using information from an area around it. Since the roughness map encodes information about sub-pixel micro-surface geometry, additional spatial resolution could be used to improve the roughness estimation. Examples of roughness textures generated by this method are shown in Figure 10.

### 4.5. Visual Results

**Figure 11:**
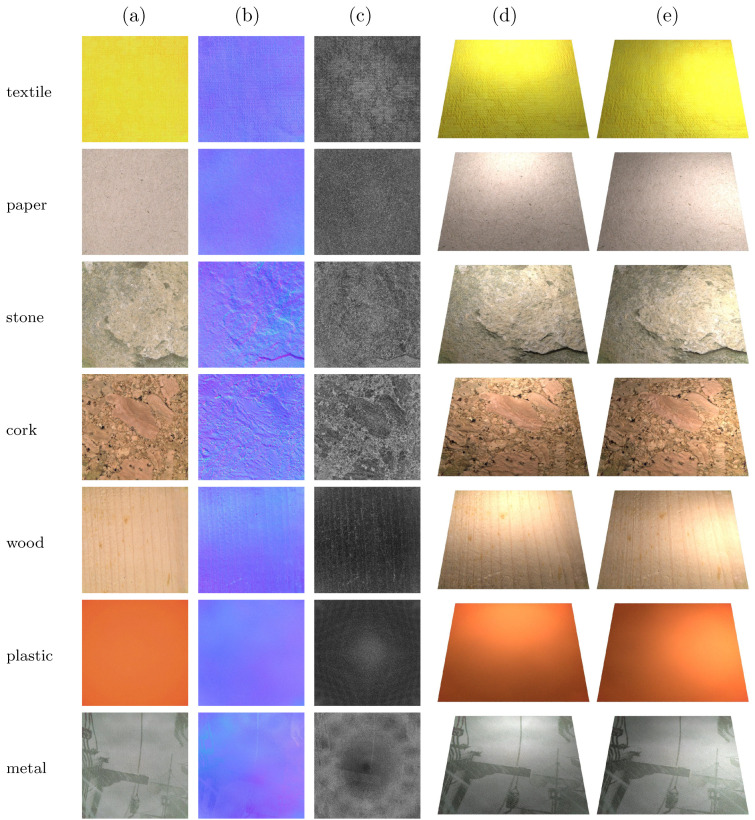
Samples of acquired roughness maps. (**a**) Picture of the surface. (**b**) Albedo texture map. (**c**) Normal texture map. (**d**) Roughness texture map. (**e**) Material re-rendering.

In Figure 11, we provide some examples of the textures acquired by the proposed device as well as re-renderings where samples are illuminated by a single strong point light. As is visible in the figure, the device captures the fine details of the three texture maps (albedo, normals, roughness). The best results are achieved on the acquisition of surfaces that present limited specular reflections such as the samples of textile, cardboard, stone, and cork. Enough details are still captured in the plastic and wood samples, which present a reflective surface. However, the printed metal sample presents some artifacts in the texture maps. In particular, it is visible how the specular reflections generated by each LED generate a normal map that represents the flat surface as a hemisphere (normals closer to the edges are slightly pointing in the direction of the edge). Given that the roughness map is generated from the normals, it highlights the presence of noise in the acquisition, and it makes visible the artifacts generated by the LEDs of the device. It is also visible in the re-rendering that the incorrect roughness does not induce specular reflections on the metal sample.

It is also worth noting that the device can correctly acquire and generate texture maps for surfaces that present strong height and normal direction variations. This is most evident in the cork and stone samples, where the former has height variations of about 1 mm and the latter of about 4 mm.

## 5. Application to Textiles Virtual Catalogs

This section discusses the use of the acquisition device in the context of a virtual catalog of textiles. The goal is to provide the user with an intuitive way to check the appearance of textiles under different lighting conditions while also being user-friendly and easy to use. To achieve this, a mobile application that uses a render engine to simulate light conditions is provided. These solutions build upon the SVBRDF maps estimated by our device.

### 5.1. Application Workflow

**Figure 12:**
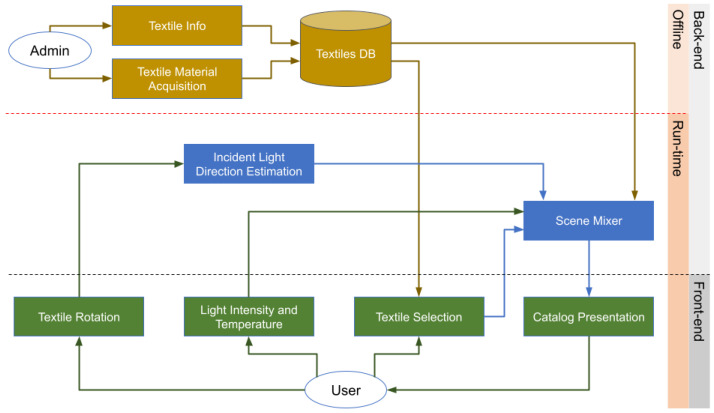
Workflow of the virtual catalog of textiles system.

Figure 12 shows the workflow of the virtual catalog application. The front-end is a mobile application, while its back-end is implemented as a cloud-based web service. The following subsections provide further details about the various components.

#### 5.1.1. Simulating the Light Interaction

**Figure 13:**
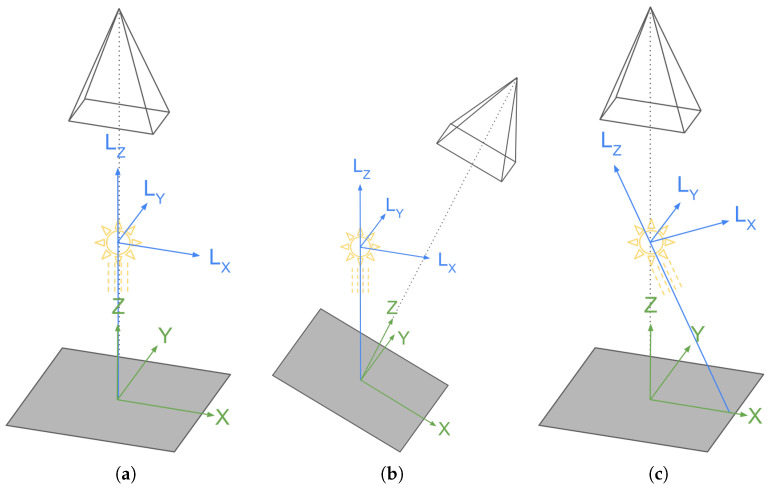
Scene setup for light interaction simulation. (**a**) Ideal conditions with view direction and incident light direction perpendicular to the sample; (**b**) real-world conditions, with the viewpoint and the sample rotating while the light source remains fixed; (**c**) a virtual setup that replicates the real-world, with the sample and viewpoint remaining fixed while the incident light direction is rotated.

The core functionality on which the virtual catalog builds is the ability to show plausible images of textiles under different light conditions. This can be achieved using a render engine. In a render engine, it is possible to set up a virtual 3D scene with the textile, a camera, and a light source, as shown in Figure 13a. In the real world, we usually check the appearance of a textile changing its orientation with respect to a light source, be it artificial or natural sunlight. Moreover, to replicate the behavior the user is used to, we should think of the screen of the device used to show the catalog as the fabric itself. Thus, by rotating the fabric (i.e., the device), the user should be able to change the angle of incident light rays (Figure 13b). This behavior can be achieved thanks to the gyroscope sensor available onboard modern smartphones and tablets.

In the 3D virtual scene, it makes more sense to change the position and orientation of the light source instead of moving the mesh used for rendering and the camera. Using a sunlight type of light source that provides uniform illumination across the whole scene, it is not necessary to change its position but only the orientation of the light rays. This results in the setup shown in Figure 13c, where the light is rotated at the same angle as the surface in Figure 13b but in the opposite direction.

The rendering of the textiles uses the Cook–Torrance-based BRDF shader model using the albedo, normal, and roughness maps provided by the acquisition device. Since the current device does not generate the specular map, the rendering uses the value 0.5, which provides a good balance between different kinds of textiles.

#### 5.1.2. User Interface

The user interface for the virtual catalog of textiles has been built as a mobile application for smartphones and tablets. The application was created using the Unity game engine [50]. Although this application is not a videogame, Unity presents features that make it possible to handle real-time rendering, data loading, and user interaction on a mobile device in a single framework. It also makes it easy to deploy the application on different mobile platforms (i.e., Android, iOS).

**Figure 14:**
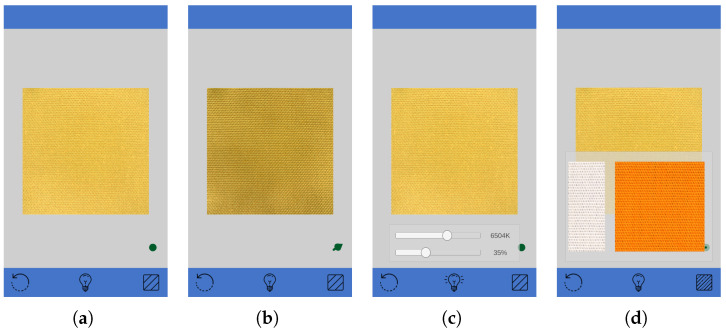
Screen captures of the virtual catalog of textiles. (**a**) Textile with perpendicular lighting; (**b**) textile with almost tangent lighting; (**c**) light setup panel; (**d**) textiles list panel.

As is visible in Figure 14, the application presents a tile of the textile as the main content. The size of the tile on the screen is set to match the real size of the acquisition.

A bottom bar with sub-panels allows the user to (from left to right) reset the tile position, customize the light’s intensity and temperature, and change the textile by choosing between previews of available textiles. Using a color picker for the light color was also investigated in the preliminary designs of the application. While it allows more freedom in the choice of the illuminant, it is also difficult for the user to make slight changes to it. On the other hand, the color temperature is a familiar concept to users and allows them to adjust the illuminant color covering the majority of real-world scenarios. By default, the temperature is set to a D65 to mimic the daylight illuminant.

The application supports touch navigation. Specifically, it is possible to resize the textile by using a pinch and translating it by using a single-finger swipe. The incident light direction is instead computed in real-time and adjusted based on the procedure described in Section 5.1.1. By changing the rotation of the device, the user can adjust the position of the sunlight providing light to the virtual scene and, thus, the final rendering of the textile. To achieve this behavior, the application uses gyroscope data to rotate the sunlight in the 3D scene. By default, the light direction is vertical above the textile, disregarding the device rotation. This initial positioning acts as a 0° rotation position to account for later changes in the device rotation. A 3D arrow pointer in the lower-right corner of the screen shows the current incident light direction. The user can force a new 0 position by touching such an arrow.

### 5.2. User Evaluation

**Figure 15:**
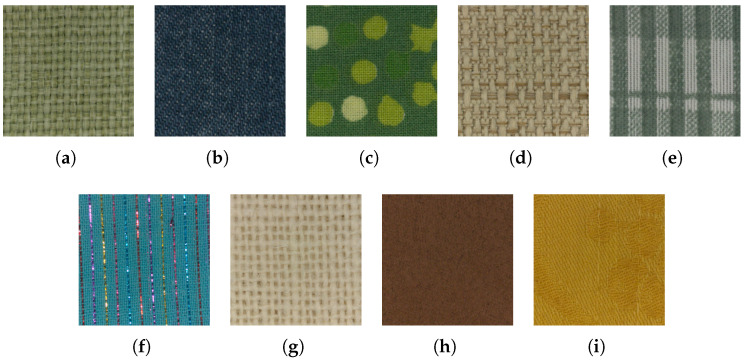
Previews of textile samples used for evaluation of the virtual catalog of textiles. (**a**) Green nylon; (**b**) blue jeans; (**c**) green cotton; (**d**) beige nylon; (**e**) printed nylon; (**f**) sparkling cotton; (**g**) white jute; (**h**) brown alcantara; (**i**) yellow damask cotton.

A standard usability test [51] with a panel of users was used to evaluate the user experience of the application. A total of 15 subjects of different ages (25–85), expertise, and educational backgrounds were selected. The experiment was conducted using a 5.5-inch Android smartphone. The same room with controlled lighting was used for all the participants. Before starting the usability test, the application and its scope were briefly described to the users. They were then observed using the application to conduct a session by consulting different textiles and their appearance changes under different light orientations, intensities, and temperatures. The actual textile for each of the samples available in the app was also provided to them to allow for direct comparison between the real and the virtual. No time limit was imposed. At the end of the test session, each user evaluated their experience by filling in a questionnaire based on the standard System Usability Scale (SUS) developed by John Brooke [52]. In order to gain more insights into the application, users were also asked to rate (from 1 to 5) the quality of the rendered textiles, the usefulness of the light interaction simulation, the usefulness of the light intensity control, the usefulness of the light temperature control, and their interest in using this application if it were made publicly available. In addition, users ranked various textiles according to the fidelity of the rendering with respect to the real textile; the same rank for multiple textiles was not allowed. The previews of samples used are visible in Figure 15. Furthermore, users were asked to explain their rationale behind the ordering chosen for the textiles. Finally, some free comments from the users were also collected.

Results of the SUS questionnaire are summarized in Table 3. Users rated the application very positively. It was considered easy and simple to use without any prior knowledge or experience. By applying the standard procedure [52], the application obtained an overall SUS score of 78 out of 100, which is considered above average. The average score is set to 68 from a study on 500 systems, as described by [53]. Table 4 shows the scores of the five additional questions specific to the textiles application. The quality of rendered textiles was highly appreciated, with a score of 4.0 out of 5. Users found the main feature of the application useful, rating its ability to simulate light interactions with a score of 4.6.

**Figure 16:**
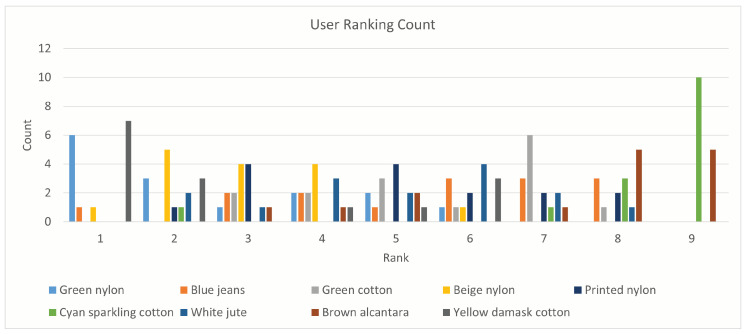
User ranking of the in-app textiles with respect to the real samples.

In general, users’ opinions are positive. The majority of them appreciated the features of the application. Users also commented on the usefulness of light interaction simulation on the textiles and how this helps to provide a more complete look and feel on a display as opposed to static images. Finally, the light controls (intensity and temperature) were appreciated. However, some users found the light intensity control less necessary than the temperature control. The ranking of the different textiles (Figure 16) shows that there is a clear opinion on which textiles are perceived as the worst and the best. However, there is no strong preference for textiles that place in the mid-field. In detail, the lowest-ranked textile samples are brown alcantara (7.3) and sparkling cotton (8.2). Their poor placement is due to missing or unsatisfactory specular reflections. The best samples are the green nylon and the yellow damask cotton (2.6) closely followed by the beige nylon (3.0). The two nylon samples (green and beige) present the same texture and material, yet the green one was consistently evaluated slightly better than the beige sample. The other samples rank close together with ranks of 5.0 for the white jute, 5.1 for the printed nylon, 5.5 for the blue jeans, and 5.7 for the green cotton. The jute sample was judged by some users to provide a plastic-looking feel. The majority of the users noticed and lamented the absence of specular reflections on the sparkling threads in the cyan textile.

Users also pointed out some problems with the application. The main concern was the presence of reflections on the screen of the smartphone when placed under a strong light source. In this scenario, the reflexes have a huge impact on the usefulness of the application since they prevent the correct perception of the rendered textile. To solve this problem, one user suggested changing the way he interacts with the application to change the incident light direction.

## 6. Conclusions

In this paper, we demonstrated the feasibility of building a low-cost, portable device for material appearance acquisition of various surfaces. Thanks to the photometric stereo technique, it has been possible to design and develop a compact device able to acquire the material properties of a limited surface area. New hardware design, software pipeline, calibration, and evaluation procedures have been defined. The device is based on consumer-grade electronics making its overall cost close to EUR 80. Quantitative and qualitative evaluations on acquired texture maps validated the system’s ability to build a correct spatially varying representation of materials with Lambertian-like reflectivity. In addition, visual results showed the capacity of the device to acquire material representations of surfaces that present non-Lambertian reflectivities, such as wood and plastics. Our evaluation of the device capabilities demonstrates that the device can be effectively exploited in Industry 4.0 applications. We also assessed this in a mobile application for the virtual catalog of textiles. The application proved to be useful in allowing people to remotely check the appearance of textiles without having the real fabric in their hands. The usability study confirmed this outcome, with users providing generally positive comments.

The current SVBRDF capture device presents some known limitations. The device has problems in the acquisition of very dark-shaded surfaces. This is due to the hardware lighting and camera setup. On such surfaces, the signal-to-noise ratio (SNR) is not sufficient to clear the noise and estimate correct normal directions using PS. This problem could be mitigated by improving the lighting of the surface with more powerful LEDs. The size of the rendered tile is limited to the size of the acquired material patch. Future work could improve this by designing a device that is able to acquire a larger patch of textiles or by generating tileable textures. At the moment, our device captures 24 images, taking about two minutes to transfer them to the server. The transfer and computational time can be reduced by optimizing the number of lights used with respect to the accuracy of the acquisition. This will also reduce the power consumption of the device. Moreover, the lights used are all identical. The device can be improved by incorporating different lights, effectively performing a multispectral acquisition of the surfaces.

Moreover, the estimation of the roughness map could be improved. Since the roughness map encodes information that regards sub-pixel micro-surface geometry, additional spatial resolution could be used to garner more insights into the micro-surface geometry and improve the estimation. Since no method for quantitative evaluation of the quality of roughness maps of real surfaces exists, defining one could be useful for the community. Providing a mapping between the physical roughness metrics and the roughness texture map could be useful to enable the evaluation. Finally, the performance and usability of the device may be further assessed in different use cases and various environmental conditions.

## Figures and Tables

**Table 1:** Comparison of the proposed device with existing approaches and devices for material acquisition.

Metric	Proposed Device	Gonioreflectometer	Multi-Camera Systems	Traditional Photometric Stereo
Accuracy (Albedo/Normals)	High (validated in tests)	Very High (lab-grade)	High	Medium to High
Cost	~€80	€10,000–€100,000+	€5000–€50,000	€1000–€5000
Portability	Highly portable	Non-portable	Limited portability	Moderate portability
Ease of Use	User-friendly	Requires expertise	Requires expertise	Moderate
Setup Complexity	Simple, compact	Complex	Complex	Moderate
References	This work	[2,5,6]	[11,37]	[4,34,35,36]

**Table 2:** Bill for the materials.

Component	Price [EUR]
Raspberry Pi Zero W	10.61
Raspberry Pi Camera Module V2	28.37
LED ring 8× SK6812 CW	1.52
LED ring 16× SK6812 CW	3.04
Camera cable	4.98
LEDs connector	0.32
LEDs flat cable	0.96
SD card 8 GB	5.03
3D printed case	26.00
*Total:*	*80.83*

**Table 3:** System Usability Scale (SUS) results.

	Statement	Strongly Disagree				Strongly Agree	Avg.	SUS Score
		1	2	3	4	5		
1	I think that I would like to use this application frequently.	0	1	3	6	5	4.0	2.6
2	I found this application unnecessarily complex.	8	4	2	1	0	1.7	3.7
3	I thought this application was easy to use.	0	1	1	4	9	4.4	3.9
4	I think that I would need assistance to be able to use this application.	8	4	2	1	0	1.7	3.9
5	I found the various functions in this application were well integrated.	0	0	1	8	6	4.3	3.3
6	I thought there was too much inconsistency in this application.	12	3	0	0	0	1.2	3.9
7	I would imagine that most people would learn to use this application very quickly.	0	0	1	6	8	4.5	3.8
8	I found this application very cumbersome or awkward to use.	10	4	1	0	0	1.4	3.6
9	I felt very confident using this application.	0	1	1	5	8	4.3	3.5
10	I needed to learn a lot of things before I could get going with this application.	11	4	0	0	0	1.3	4.0

**Table 4:** Application-specific question results.

Question	Average Rating
Quality of rendered textiles	4.0
Usefulness of light interaction simulation	4.6
Usefulness of light temperature control	4.5
Usefulness of light intensity control	4.4
Would use the application	4.2

## Data Availability

Samples of acquired data shown in this article will be made available by the authors on request.

## Abbreviations

The following abbreviations are used in this manuscript:

BRDF	Bidirectional reflectance distribution function
GPIO	General-purpose input/output
LED	Light-emitting diode
PS	Photometric stereo
SVBRDF	Spatially varying bidirectional reflectance distribution function
